# Acute inorganic nitrate ingestion does not impact oral microbial composition, cognitive function, or high-intensity exercise performance in female team-sport athletes

**DOI:** 10.1007/s00421-024-05552-w

**Published:** 2024-07-17

**Authors:** Rachel Tan, Courtney Merrill, Chandler F. Riley, Maya A. Hammer, Ryan T. Kenney, Alyssa A. Riley, Jeffrey Li, Alexandra C. Zink, Sean T. Karl, Katherine M. Price, Luka K. Sharabidze, Samantha N. Rowland, Stephen J. Bailey, Leah T. Stiemsma, Adam Pennell

**Affiliations:** 1 Natural Science Division, Pepperdine University, Malibu, CA 90263 USA; 2 School of Sport, Exercise and Health Sciences, Loughborough University, Loughborough, UK

**Keywords:** Nitric oxide, Beetroot, Exercise, Strength, 16S rRNA sequencing, Females

## Abstract

The purpose of this study was to investigate the effects of acute nitrate (NO_3_^−^)-rich beetroot juice ingestion on explosive and high-intensity exercise performance, oral microbiota composition, and cognitive flexibility (i.e., function), before and after maximal intermittent running exercise. Fifteen women team-sport athletes were assigned in a randomized, double-blind, crossover design to consume concentrated NO_3_^–^-depleted beetroot juice (PL; 0.1 mmol NO_3_^−^) and NO_3_^−^-rich beetroot juice (BR; 12.0 mmol NO_3_^−^) 2.5 h prior to performing a battery of exercise performance tasks and cognitive testing before and after the Yo–Yo intermittent recovery level 1 (YYIR1) running test. Resting plasma [NO_3_^−^] and plasma nitrite ([NO_2_^−^]) were elevated following BR (*P* < 0.001). BR did not impact global composition or relative abundance of taxa in the oral microbiome (*P* > 0.05) or cognitive flexibility before or after exercise (*P* > 0.05). There was no significant difference in performance during 20-m (PRE, PL: 4.38 ± 0.27 vs. BR: 4.38 ± 0.32 s; POST, PL: 4.45 ± 0.29 vs. BR: 4.43 ± 0.35 s) and 10-m sprints (PRE, PL 2.78 ± 0.15 vs. BR 2.79 ± 0.18 s; POST, PL: 2.82 ± 0.16 vs. BR: 2.81 ± 0.19 s), isokinetic handgrip dynamometry, medicine ball throw, horizontal countermovement jump, or YYIR1 (PL: 355 ± 163 m vs. BR: 368 ± 184 m) between BR and PL (*P* > 0.05). These findings indicate that acute dietary NO_3_^−^ may not influence the oral microbiome, explosive and high-intensity exercise performance, or cognitive function in women team-sport athletes.

**Supplementary Information:**

The online version contains supplementary material available at 10.1007/s00421-024-05552-w.

## Introduction

Dietary nitrate (NO_3_^−^) is a purported ergogenic aid with the potential to enhance exercise performance in multiple exercise modalities, such as running, cycling, and weightlifting (Senefeld et al. 2020; Tan et al. 2023). The ergogenic effects of dietary NO_3_^−^ are thought to be mediated by elevating nitric oxide (NO), a key signaling molecule that regulates numerous physiological processes (Stamler & Meissner 2001). The metabolism of exogenous NO_3_^−^ to nitrite (NO_2_^−^) is facilitated by NO_3_^−^-reducing oral microbial species during second-pass NO_3_^−^ metabolism (Lundberg et al. 2008). Subsequent enzymatic (Millar et al. 1998) and non-enzymatic (Shiva et al. 2007) reactions reduce NO_2_^−^ to NO, particularly in hypoxic (Castello et al. 2006) and acidic tissues (Modin et al. 2001). It is well documented that plasma [NO_2_^−^] is elevated following NO_3_^−^ supplementation (Wylie et al. 2013a) and the magnitude of this increase appears to be important for eliciting ergogenic effects (Coggan et al. 2018; Porcelli et al. 2015; Wilkerson et al. 2012). Recent data also suggest that exogenous NO_3_^−^ can increase muscle [NO_3_^−^] with both the magnitude of increase in muscle [NO_3_^−^] prior to exercise and the decline in muscle [NO_3_^−^] during exercise positively associated with performance during maximal muscle contractile performance (Kadach et al. 2023).

The determinants of performance in team-sports are multifactorial but include the ability to sprint and accelerate linearly and in multiple directions in response to rapid decision-making during competition (Haugen et al. 2019b). Sprinting performance is a function of maximal horizontal power output and velocity (Haugen et al. 2019a), rate of force development (Aagaard et al. 2002), and muscular strength (Andersen & Aagaard 2006), all of which are performance outcomes that heavily rely on type II muscle fiber recruitment (Krustrup et al. 2006; Morton et al. 2019). In rodent models, dietary NO_3_^−^ supplementation has been shown to preferentially alter physiological responses in type II muscle fibers, including improving blood flow (Ferguson et al. 2013) and contractile function via augmented intramyocyte calcium handling (Hernández et al. 2012). These positive effects on type II muscle fibers after NO_3_^−^ supplementation in murine models are thought to underpin the small improvements in single sprint and high-intensity intermittent exercise performance (Alsharif et al. 2023), and power (Coggan et al. 2021; Tan et al. 2023), and strength (Lago-Rodríguez et al. 2020), after NO_3_^−^ supplementation in humans. However, while there is evidence to support the ergogenic potential of NO_3_^−^ supplementation in high-intensity exercise (Tan et al. 2022), most studies to date have been conducted on male participants (Wickham et al. 2019). Men and women may have divergent capacities for NO synthesis and/or NO_3_^−^ and NO_2_^−^ storage due to differences in muscle fiber type (Wickham & Spriet 2019), NO synthase expression (Hickner et al. 2006), salivary flow rate (Inoue et al. 2006), and oral microbial species/activity (Inoue et al. 2006; Kapil et al. 2018). Moreover, storage sites for NO_3_^−^ include muscle mass (Wylie et al. 2019) and the surface area of skin (Fujii et al. 2023), but sex differences in these body compartments (Dao & Kazin 2007; Janssen et al. 2000) may influence the storage and metabolism, and thus, the physiological effects of NO_3_^−^. Therefore, further empirical investigation is required to determine whether dietary NO_3_^−^ supplementation improves exercise performance (i.e., power, endurance, strength, and total work) specifically in women.

To date, a few studies have investigated the influence of NO_3_^−^ supplementation on reliable and valid field-based protocols, such as the Yo–Yo intermittent recovery level 1 test (YYIR1) (Bangsbo et al. 2008), that reflect intermittent exercise performance during team-sport match play (i.e., soccer and rugby) (Krustrup et al. 2005). This shortcoming limits the application of findings to real-world sports. Of the limited available data, most (Esen et al. 2022; Nyakayiru et al. 2017; Thompson et al. 2016), but not all studies (Esen et al. 2023) in males, have reported enhanced YYIR1 performance after NO_3_^−^ supplementation, but the effects after acute NO_3_^−^ ingestion are less clear (Esen et al. 2022, 2023). Importantly, the effects of NO_3_^−^ supplementation on YYIR1 performance has not been assessed in women. While some initial evidence suggested that power output could be increased in women after NO_3_^−^ supplementation (Coggan et al. 2018), the limited current evidence suggests that there are no effects of NO_3_^−^ supplementation in single or repeated sprints (López-Samanes et al. 2022, 2023), strength (López-Samanes et al. 2022, 2023), endurance (Ortiz de Zevallos et al. 2023), power (Poredoš et al. 2022), and economy (Forbes & Spriet 2021; López-Samanes et al. 2023; Ortiz de Zevallos et al. 2023; Poredoš et al. 2022) and could perhaps compromise performance (Hogwood et al. 2023). Furthermore, cognitive function is critical during team-sport match play for decision-making and reactions (Vestberg et al. 2012) and NO_3_^−^ supplementation has been reported to improve reaction time (Thompson et al. 2016; Wightman et al. 2015). However, the effects of NO_3_^−^ on other domains of cognitive performance, such as cognitive flexibility (i.e., creativity, complex problem solving, adaptability, etc. [Diamond 2013]) have yet to be comprehensively explored. Collectively, since less is known regarding high-intensity exercise in women, further research is required.

There is increasing interest in role of symbiotic oral microbiota on NO_3_^−^ metabolism and thus the efficacy of NO_3_^−^ supplementation to improve physiology and performance (Jones et al. 2021). Of the limited available data, multi-day NO_3_^−^ supplementation was shown to shift the microbial composition into health-associated and NO_3_^−^-reducing bacteria (Rosier et al. 2020; Vanhatalo et al. 2018, 2021). However, the effects of NO_3_^−^ supplementation on the oral microbiome are currently limited, particularly in women, and it is unknown if other supplementation regimens such as acute NO_3_^−^ ingestion can elicit similar effects.

The purpose of this study was to examine the influence of acute NO_3_^−^ ingestion on the oral microbiome composition, and a battery of exercise performance and cognitive function tasks before, during, and after the YYIR1, in women team-sport players. It was hypothesized that, compared to NO_3_^−^-depleted beetroot juice, an acute dose of NO_3_^−^-rich beetroot juice would alter the oral microbiome composition to promote NO_3_^−^-reducing taxa and improve exercise and cognitive performance before, during, and following the YYIR1.

## Materials and methods

### Participants

Fifteen women team-sport athletes from intramural and University teams (mean ± SD, age: 20 ± 1 years; body mass: 63 ± 10 kg; height: 1.68 ± 0.1 m; and $\dot{\text{V}}$ O_2peak_: 35 ± 5 mL·kg^−1^·min^−1^) volunteered to participate in this study following a power calculation based on a previously published report (Nyakayiru et al. 2017) using an effect size *d*_*z*_ of 1.03, power of 0.95 and alpha of 0.05. All participants were of Caucasian race and were given a random identification code for anonymization. The protocols, risks, and benefits of participating were explained prior to obtaining written informed consent and participants completed a screening and a physical activity readiness questionnaire. This study was registered on the Open Science Framework database (osf.io/rsxep) on 28 June 2023, and was approved by the Institutional Research Ethics Committee and conformed to the code of ethics of the Declaration of Helsinki.

Experimental data collection for this study was not timed according to a particular phase within the menstrual cycle due to logistical, time, equipment, and financial challenges; however, all women included in this study were defined as naturally menstruating (menstrual cycle length ≥ 21 and ≤ 35 days in duration) (Elliott-Sale et al. 2021). Moreover, it is important to note that controlling for hormonal fluctuations across the menstrual cycle may reduce the external validity of study findings (Stanhewicz & Wong 2020) and it is still unclear if the physiological responses to NO_3_^−^ supplementation are influenced by menstrual cycle phase (Baranauskas et al. 2022; Smith-Ryan et al. 2022). The participant exclusion criteria were individuals with contraindications to exercise, cardiometabolic disease, on recreational supplementation, women on birth control, women with oral diseases, men, and smokers. Women on birth control were excluded given that hormonal contraceptives may impact the interaction between sex hormones and skeletal muscle contraction (Sarwar et al. 1996). Men were excluded given that limited data exist in exclusively women cohorts in dietary NO_3_^−^ research and the current study aimed to examine women as an underrepresented population (Wickham et al. 2019).

### Experimental overview

**Fig. 1:**
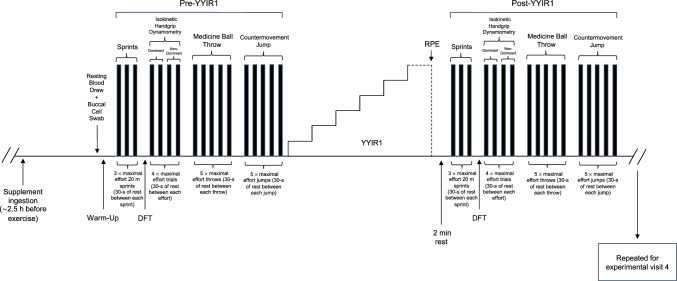
Overview of experimental protocol. YYIR1 = Yo–Yo intermittent recovery level 1 running test; pre-YYIR1 = before Yo–Yo intermittent recovery level 1 test; post-YYIR1 = after Yo–Yo intermittent recovery level 1 test; DFT = design fluency test (i.e., cognitive flexibility); RPE = rate of perceived exertion

Participants reported to the laboratory on 4 separate occasions over a 4-week period. During visit 1, participants completed a ramp incremental cycling exercise test for the determination of $\dot{\text{V}}$ O_2peak_. During visit 2, participants were familiarized to the experimental testing procedures, including the completion of 20-m sprints, YYIR1 (Krustrup et al. 2003), testing of cognitive performance via assessment of cognitive flexibility (Delis et al. 2001), maximal handgrip strength (National Institutes of Health [NIH] & Northwestern University 2018), and explosive strength tasks (Sharp et al. 2018). Subsequently, in a double-blind, randomized, crossover design, participants were assigned to two experimental conditions using a web-based randomizer (random.org) to receive either 2 × 70 ml of concentrated NO_3_^−^-depleted placebo (PL; 0.10 mmol NO_3_^−^ total) or NO_3_^−^-rich beetroot juice (BR; ~ 12.0 mmol NO_3_^−^ total) with a washout-out period of at least 5 days separating the two supplementation periods. The experimental protocol was completed on the subsequent experimental visits (i.e., visit 3 and 4) and is displayed in Fig. 1. During the experimental protocol, participants performed a battery of exercise procedures prior to a YYIR1 test (i.e., pre-YYIR1) which included 20-m running sprints, cognitive performance testing (i.e., cognitive flexibility), isokinetic handgrip dynamometry, seated medicine ball throws, and horizontal countermovement jumps, followed by the YYIR1 test. After 2 min of recovery, participants performed the same battery of exercise procedures (i.e., post-YYIR1 and in the same order). The experimental protocol was used to determine sprint times/splits, sprint initiation response time (i.e., summed movement and reaction time following a randomized visuo-auditory stimulus), cognitive performance, maximal handgrip strength, and upper and lower body explosive strength, prior to, and following completion of the YYIR1, as well as YYIR1 performance and RPE.

### Supplementation procedures

During the experimental visits (i.e., visit 3 and 4), participants consumed 2 × 70 mL of their allocated beverage ~ 2.5 h prior to the exercise given that this timing is associated with the attainment of peak values for plasma [NO_3_^−^] and [NO_2_^−^] (Wylie et al. 2013a) of BR (~ 5.9 mmol of NO_3_^−^ per 70 mL; Beet It Sport, James White Drinks Ltd., Ipswich, UK) or PL (~ 0.05 mmol of NO_3_^−^ per 70 mL; Beet It Sport, James White Drinks Ltd., Ipswich, UK). Each 70 mL beetroot juice beverage contained 72 kcal energy and 15.4 g of carbohydrate. The randomization, allocation, and concealment of the beverages were conducted by a researcher that was not involved in data collection or data analysis procedures, to ensure that the main researchers and participants remained blinded to the conditions (i.e., double-blinded). The beverages were identical in taste, appearance, and smell; with the NO_3_^−^ ions removed by an ion-exchange resin to create the PL drink (Gilchrist et al. 2014). For the duration of the study, participants were asked to maintain their habitual physical activity and dietary intake. Participants recorded their activity and diet during the 24 h prior to the first experimental visit and were asked to repeat these for subsequent visits. To ensure compliance: (1) participants were informed the importance of adhering to the lifestyle behavior instructions during screening; (2) researchers sent text message reminders throughout the 24 h prior to each experimental visit and; (3) at the start of each visit, participants provided written and verbal confirmation that their physical activity and dietary habits were identical across visits. Researchers provided a list of foods high in dietary nitrate NO_3_^−^ (i.e., beetroot, kale, spinach, and arugula) and dietary supplements to avoid consuming, such as sodium bicarbonate, creatine, beta-alanine, and/or precursor supplements (i.e., NO_3_^−^, arginine, citrulline, and antioxidants) as well as to refrain from brushing their teeth on laboratory visits. Participants were instructed to avoid using antibacterial mouthwash and chewing gum for the duration of the study given that it has been evidenced to interfere with NO_3_^−^ metabolism in humans (Govoni et al. 2008). Experimental visits were performed at the same time of day (± 1 h). Participants were instructed to arrive at the laboratory having avoided strenuous exercise and alcohol in the 24 h preceding, and caffeine in the 8 h preceding, each experimental visit.

### Measurements

#### Maximal aerobic capacity

On visit 1, participants performed an incremental ramp test on an electronically braked cycle ergometer (Excalibur Sport, Lode, The Netherlands), involving 3 min of baseline cycling at 20 W, followed by a work rate of 30 W/min until task failure (i.e., when pedaling cadence fell by > 10 rpm below the self-selected cadence of 70–90 rpm). Breath-by-breath pulmonary gas exchange and ventilation was measured continuously and averaged over consecutive 30-s periods during the incremental test. Participants wore a face mask and mask adapter with a headstrap (7450 V2 series, Hans Rudolph, USA) and breathed through a low dead space (99 ml) pitot tube flow sensor assembly. The inspired and expired gas volume and gas concentration signals were continuously sampled at 100 Hz using electrochemical (oxygen) and infrared (carbon dioxide) analyzers (Ultima™ CardiO2® Gas Exchange Analysis System, MGC Diagnostics Corporation, USA) via a capillary line attached to the face mask. Calibration of gases and volume was conducted prior to each test using gases of known concentration and a 3-L syringe (Hans Rudolph, Kansas City, MO). The volume and concentration signals were time aligned by accounting for the delay in capillary gas transit and analyzer rise time relative to the volume signal. The analyzer used standard formulas (Beaver et al. 1981) to calculate the volume of oxygen, carbon dioxide, and minute ventilation and displayed as breath-by-breath.

#### *Plasma [NO*_*3*_^*−*^*] and [NO*_*2*_^*−*^*] analysis*

A resting venous blood sample was obtained from the antecubital vein of the forearm by a phlebotomy trained member of the research team upon arrival to the laboratory for the assessment of plasma [NO_3_^−^] and [NO_2_^−^]. Samples were drawn into 6-mL lithium heparin tubes (Vacutainer, Becton–Dickinson, New Jersey, USA) and centrifuged at 3100 × g at 4 °C for 10 min within 2 min of collection. Plasma was extracted and stored in a − 80 °C freezer for the analysis of plasma NO_3_^−^ and NO_2_^−^ using gas phase chemiluminescence. All glassware, utensils, and surfaces were rinsed with deionized water to remove NO_3_^−^ and NO_2_^−^ prior to analysis. Plasma samples were thawed and then deproteinized using ice-cold ethanol precipitation prior to [NO_2_^−^] analysis. Specifically, samples were centrifuged at 14,000 × g for 10 min, and 200 μL of the supernatant was treated with 400 μL of ice-cold ethanol. Samples were then incubated on ice for 30 min, and subsequently centrifuged at 14,000 × g for 10 min. The [NO_2_^−^] of deproteinized plasma was determined by its reduction to NO using glacial acetic acid and aqueous sodium iodide and calibrated using sodium NO_2_^−^ standards. Following this, the deproteinized plasma samples were diluted prior to [NO_3_^−^] analysis, such that 100 μL of the supernatant was added to 400 μL of deionized water. The [NO_x_] (i.e., NO_3_^−^ + NO_2_^−^) of diluted deproteinized plasma was determined by its reduction to NO using vanadium chloride and hydrochloric acid and calibrated using sodium NO_3_^−^ standards. Subsequently, the [NO_2_^−^] values were subtracted from [NO_x_] to obtain [NO_3_^−^] values.

#### Oral microbiome

A resting oral buccal cell sample was obtained ~ 2.5 h post-ingestion of BR and PL, using cotton swabs and stored in a DNA shield buffer (Zymo, Zymo Research Incorporation, Irvine, USA) in a -80 °C freezer until later analysis of the microbiome. Samples were thawed, and DNA was extracted using the Isohelix Buccal-Prep Plus DNA Isolation Kit (Isohelix, Cell Projects Ltd., Dedham, USA) according to the manufacturer’s guidelines. Specifically, samples were lysed by vortexing the sample with 20 µL of proteinase K and 500 µL of BLS solution before being placed in a 60 °C water bath for 1 h. Following buccal cell lysis, the samples were centrifuged and washed with BP solution and eluded in 50 µL of TE solution. Double-stranded DNA concentration was quantified using the Nanodrop spectrophotometer (Nanodrop UV–Vis, ThermoFisher Scientific, USA). Isolated DNA was stored at -80 °C until library preparation and sequencing. Amplification of bacterial 16S ribosomal DNA was performed using polymerase chain reaction (PCR) primers targeting the 16S rRNA gene V3-V4 (319F and 806R). The samples were purified with the QIAquick PCR Purification Kit (Qiagen, Maryland, USA) and indexed using the Nextera XT DNA Library Prep Kit (Illumina, CA, USA). Following a final purification with the QIAquick PCR Purification Kit (Qiagen, Germany), the purified samples were quantified via a Qubit Assay (ThermoFisher Scientific, USA), pooled, and sequenced on the Illumina iSeq System (150 × 150 paired-end sequencing, Illumina, CA, USA).

Raw sequence reads were preprocessed into amplicon sequence variants (ASVs) using DADA2 (Callahan et al. 2016). We followed the standard DADA2 tutorial with the following modifications: for the filter_and_trim function, we trimmed forward and reverse sequence reads to 149 bp, for the mergePairs function, justConcatonate was set to TRUE. Contaminant ASVs were removed using the R package, decontam, based on the prevalence of reads in 1 negative control samples (Davis et al. 2018). Samples with less than 1000 reads were removed from analysis. The APE package in R was used to construct a phylogenetic tree (Paradis & Schliep 2019). All samples were rarefied to the same read depth of 2223 reads for alpha and beta diversity analysis (reducing the number of samples to 27). The unrarefied data set was used for relative abundance analysis. Analysis of alpha and beta diversity and relative abundance was conducted using the Phyloseq package in R (McMurdie & Holmes 2013). All R code for this analysis can be found in Online Resource 1–3. Raw sequence reads are deposited in in the NCBI Sequence Read Archive (SUB14279269).

#### Sprinting performance

On experimental visits, all exercise was performed on a wooden surface in an indoor sports hall, and all running protocols (i.e., YYIR1 and 20-m sprints) were performed in running lanes of 2 × 20-m lanes marked by cones as previously described (Krustrup et al. 2003). Participants performed a standardized warm-up prior to the experimental protocol consisting of a 3-min jog, dynamic stretches, 2 × 10-m backwards sprints at 75% and 90% perceived maximal effort, and 2 × 20-m sprints at 75% and 90% perceived maximal effort starting from a three-point stance. Following this, based on a previous study (Thompson et al. 2016), participants began in a stationary three-point-stance position and performed three maximal running sprints over a distance of 20 m that was interspersed with 30 s of active walking recovery. A timing gate system (Smartspeed, Fusion Sports, Australia) was positioned at 0, 10, and 20 m and provided a randomly timed (1- to 5-s foreperiod) and simultaneous visual (green lights) and auditory (beep) stimuli to signal the start of each sprint. Response time and split times were recorded at each timing gate.

#### Cognitive performance

Immediately following the sprints, participants completed the Delis-Kaplan Executive Function System design fluency test (DFT) (Delis et al. 2001) which is a non-verbal, psychomotor, norm-referenced cognitive flexibility test. The DFT has previously been used in team-sport athletes and has been shown to be predictive of sporting success (Vestberg et al. 2012, 2017).

Using standardized paper templates containing rows of boxes with arrays of dots and a pen, participants were instructed to create as many unique designs as possible within 1 min of time using four connected and straight lines. For each test, after an initial practice round, this process was repeated across three pre-determined conditions: filled dots (condition 1), empty dots (condition 2), and switching (condition 3). Subsequently, raw scores (i.e., the number of correct/unique designs) for each condition were converted to individual scaled scores. The three scaled scores for each condition were then summed to create a summed scale score. The summed scale scores were then converted to a composite scaled score which was used as the variable of analysis. Composite scaled scores ranged from 1 to 19 with higher scores representing better cognitive flexibility.

#### Maximal strength

Immediately following cognitive flexibility testing, participants performed two seated trials (one practice/submaximal; one test/maximal trial) of isokinetic handgrip dynamometry via a Jamar® Smart Digital Hand Dynamometer (Performance Health, Warrenville, IL) for their dominant hand, followed by their non-dominant hand, interspersed by 30 s of rest (NIH & Northwestern University 2018), to assess maximal strength (Wind et al. 2010). As per established protocol guidelines (NIH & Northwestern University 2018), handgrip testing occurred with the handle in second position, forearms in a neutral position, and the active arm/elbow flexed 90°. Participants were encouraged by the examiner who provided standardized verbal encouragement during the maximal trials which lasted 3–4 s. The maximum value (kg) from each hand was retained for analysis.

#### Explosive strength

Immediately following isokinetic handgrip dynamometry, participants performed assessments for upper and lower body explosive strength, in the horizontal plane, by completing seated medicine ball throws and countermovement jumps, respectively, which were based on the Occupational Physical Assessment Test (Sharp et al. 2018). For the seated medicine ball throws, participants were instructed to sit against a wall and to bring a 2 kg medicine ball to their chest, and then to extend their arms at a 45° angle using maximal effort to throw the medicine ball for five throws, each separated by 30 s of recovery, with the maximum distance recorded to the nearest tenth of a meter. For the standing long jumps, participants were instructed to begin in a stationary position, and then to swing their arms backwards, to bend their knees, and then to propel forward as far and quickly as possible for five jumps, each separated by 30 s of recovery. Participants were required to land with both feet and without falling backwards, and the furthest distance (i.e., heel closest to the take-off line) was recorded to the nearest hundredth of a meter.

#### YYIR1 performance

Based on a previous validation study (Krustrup et al. 2003), and previous nitrate research (Nyakayiru et al. 2017; Wylie et al. 2013a), the YYIR1 consisted of 2 × 20-m sprints, indicated by audio bleeps that increased in speed with each level. Participants had a 10-s active recovery period between sprints in a 5 × 2-m area marked by cones behind the starting line. The distance was recorded when a participant failed to reach the finishing line before the audio bleeped twice. Immediately upon completion of the YYIR1, the ratings of perceived exertion (RPE) were measured and recorded using a Borg scale from 6 to 20 (Borg 1982). Following 2 min of recovery after the YYIR1, participants repeated the battery of tests performed pre-YYIR1 which consisted of 20-m sprint efforts, DFT, isokinetic handgrip dynamometry, seated medicine ball throws, and countermovement jumps.

### Statistical analyses

Two-way repeated-measures ANOVAs (condition × time) were used to analyze differences in performance for sprint-related times, DFT composite scaled scores, isokinetic handgrip strength, seated medicine ball throw, and countermovement jumps with Bonferroni corrections when applicable. Differences in plasma [NO_3_^−^] and [NO_2_^−^], distance covered, and RPE during the YYIR1 were analyzed using paired *t* tests. Unless stated otherwise, all statistical assumptions were met (e.g., normality of the residuals, sphericity). Effect sizes for ANOVAs were measured via partial eta-squared (*η*_*p*_^2^) in which small, medium, and large effects were operationalized as 0.01, 0.06, and 0.14, respectively (Cohen 1988). Effect sizes for *t* tests were measured as Cohen’s *d*_*z*_ in which small, medium, and large effects were operationalized as 0.2, 0.5, and 0.8, respectively (Cohen 1988; Lakens 2013). For microbiome analysis, statistical comparisons across groups for beta diversity were determined using the betadisper test and PERMANOVA (McMurdie & Holmes 2013; Oksanen et al. 2024). Statistical comparisons across groups for alpha diversity measures were determined using the Shapiro–Wilk test for normality (*P* ≤ 0.05 means data is normally distributed) and ANOVA in R. We used MaAsLin2 under default settings (*q* value threshold of 0.25) to identify differentially abundant ASVs (Mallick et al. 2021). Statistical significance was set to *P* ≤ 0.05 with all data presented as mean ± SD. All data were analyzed using SPSS version 27 (IBM, Armonk NY).

## Results

All participants reported consuming all servings of each supplement at the correct times and verbally confirmed that they had maintained their exercise and dietary habits prior to each testing visit. Furthermore, all participants verbally confirmed that they did not notice any differences between supplements. There were two reports of gastrointestinal distress (i.e., mild-to-moderate nausea) immediately following the ingestion of supplements.

### Plasma [NO3−] and [NO2−]

Plasma [NO_3_^−^] and plasma [NO_2_^−^] results are displayed in Table 1. The coefficient of variation for duplicate samples was 1.5 ± 0.3% and 9.1 ± 10.3% for plasma [NO_3_^−^] and [NO_2_^−^], respectively. Plasma [NO_3_^−^] was higher in BR compared to PL (*P* < 0.001, *d*_*z*_ = 4.49). Plasma [NO_2_^−^] was higher in BR compared to PL (*P* < 0.001, *d*_*z*_ = 2.01).

**Table 1:** Indices of nitric oxide bioavailability following acute nitrate ingestion

Variable	PL	BR
Plasma [NO_3_^−^] (µM)	52 ± 14	629 ± 132^***^
Plasma [NO_2_^−^] (nM)	276 ± 286	703 ± 391^***^

BR, nitrate-rich beetroot juice; NO_3_^−^, nitrate; NO_2_^−^, nitrite; PL, nitrate-depleted beetroot juice; µM, micromolar; nM, nanomolar

^***^P ≤ 0.001 (significantly different compared to placebo)

### Oral microbiome

**Fig. 2:**
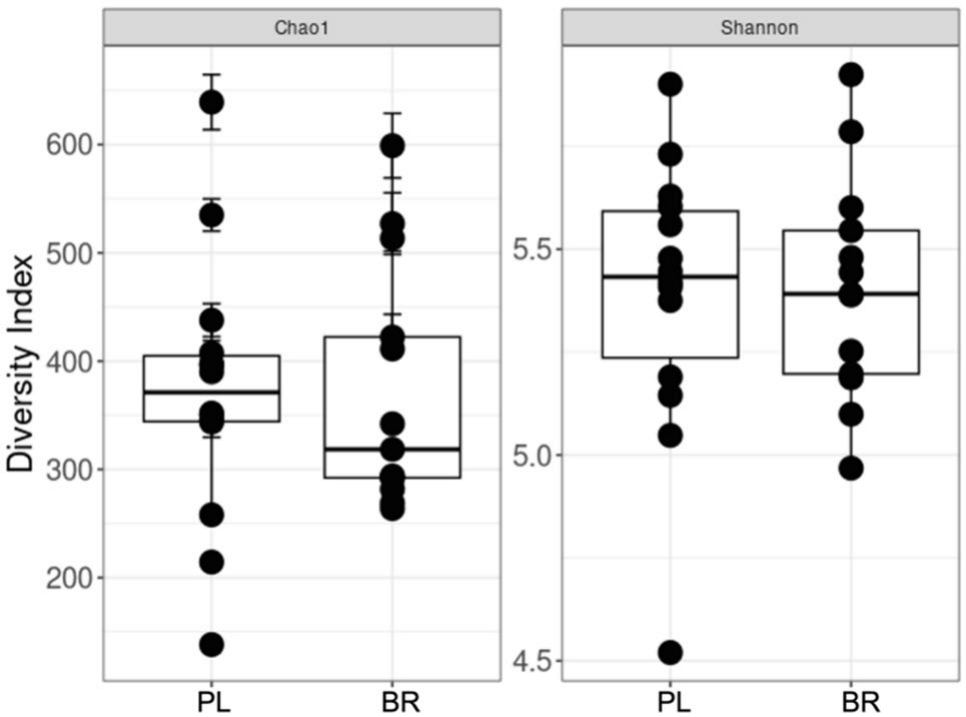
No differences in Alpha diversity (Chao1 and Shannon diversity index) of the oral microbiome in PL and BR

**Fig. 3:**
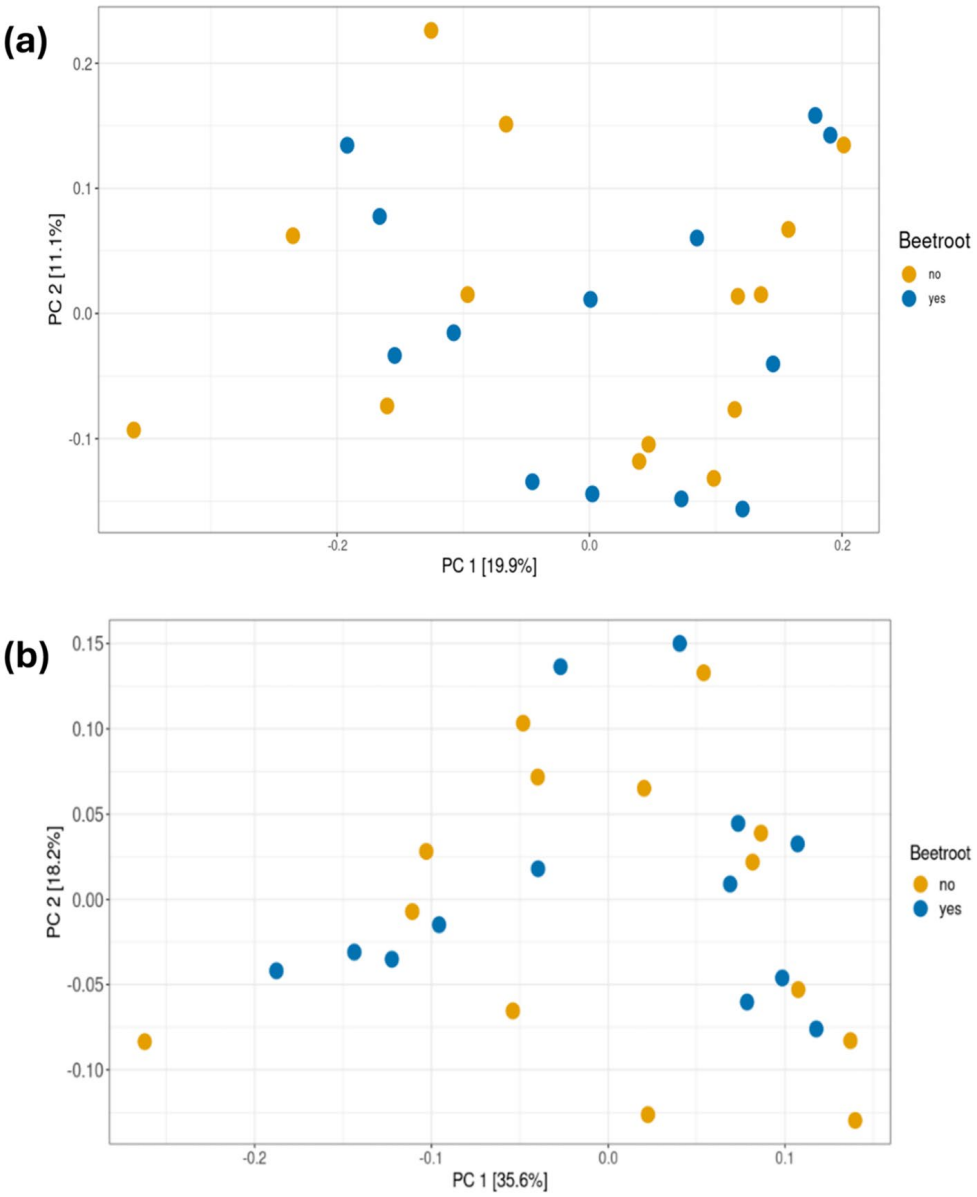
No significant clustering in unique fraction metric (UniFrac) principal coordinates analysis (PCoA) in PL and BR displayed as **a** unweighted and **b** weighted

**Fig. 4:**
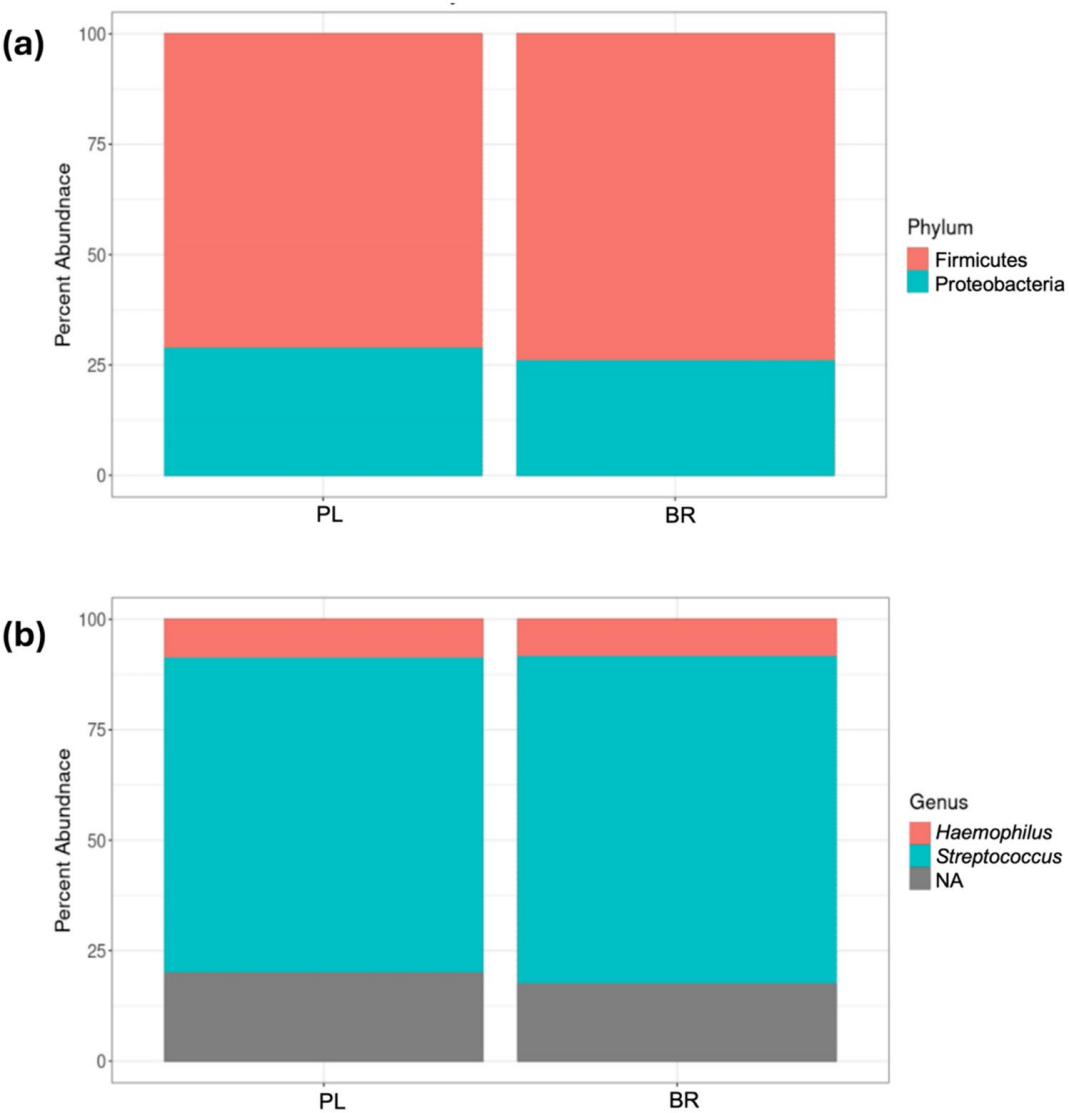
No differences in PL and BR based on the top 100 amplicon sequence variants in **a** phylum relative abundance and **b** family relative abundances

An insufficient amount of DNA was present in one sample, and thus, data for a subset of participants (*n* = 14) are presented. Alpha diversity was analyzed using the Shannon diversity (species richness and evenness) and Chao1 (species richness) indexes and no differences were observed between PL and BR (Fig. 2). Beta diversity was analyzed via principal component analysis (PCoA) and no significant clustering was observed (Fig. 3a, b). There were no differences in relative abundance of the phylum or genus (*P* > 0.05) (Fig. 4a, b).

### Sprint performance

There was no interaction (condition x time) or main effect of condition on 10-m and 20-m split sprint times (*P* > 0.05, Table 2.). There was an effect of time, such that split times increased for 10-m (*P* = 0.017, *η*_*p*_^*2*^ = 0.35) and 20-m post-YYIR1 (*P* = 0.002, *η*_*p*_^*2*^ = 0.52). There was no interaction effect (condition x time) or main effects of condition or time on sprint response time (*P* > 0.05, Table 2.).

**Table 2:** Sprint performance before and after the YYIR1 test following acute beetroot juice ingestion

Variable	PL		BR	
	Pre	Post	Pre	Post
Response time (s)	0.57 ± 0.10	0.55 ± 0.12	0.52 ± 0.09	0.55 ± 0.11
10-m split time (s)	2.78 ± 0.15	2.82 ± 0.16^**^	2.79 ± 0.18	2.81 ± 0.19^**^
20-m split time (s)	4.38 ± 0.27	4.45 ± 0.29^**^	4.38 ± 0.32	4.43 ± 0.35^**^

BR, nitrate-rich beetroot juice; PL, nitrate-depleted beetroot juice

^**^P ≤ 0.01 (significantly different to pre-YYIR1)

### Cognitive performance

There was no interaction effect (condition x time) or main effect of condition on the DFT composite scale score (*P* > 0.05, Table 3.). There was a main effect of time, such that scores before the YYIR1 (PL: 15.3 ± 2.7 vs. BR: 15.8 ± 2.7) increased after the YYIR1 in both conditions (PL: 16.9 ± 1.4 vs. BR: 16.6 ± 1.9, *P* = 0.010, *η*_*p*_^*2*^ = 0.39).

**Table 3:** DFT composite scale score performance before and after the YYIR1 test following acute beetroot juice ingestion

Variable	PL		BR	
	Pre	Post	Pre	Post
DFT	15.27 ± 2.71	16.93 ± 1.44 ^**^	15.80 ± 2.65	16.60 ± 1.88 ^**^

BR, nitrate-rich beetroot juice; PL, nitrate-depleted beetroot juice; DFT, Design Fluency test

^**^P ≤ 0.01 (significantly different to pre-YYIR1)

### Maximal strength

There was no interaction effect (condition x time) or main effect of condition or time on maximal strength as assessed by isokinetic handgrip dynamometry in the dominant and non-dominant hand (*P* > 0.05, Table 4).

**Table 4:** Maximal isokinetic handgrip dynamometry strength before and after the YYIR1 test following acute beetroot juice ingestion

Variable	PL		BR	
	Pre	Post	Pre	Post
Dominant handgrip strength (kg)	34.64 ± 4.74	34.66 ± 6.06	35.27 ± 5.68	35.54 ± 5.59
Non-dominant handgrip strength (kg)	31.75 ± 5.21	31.62 ± 5.30	32.13 ± 5.24	32.07 ± 6.01

BR, nitrate-rich beetroot juice; PL, nitrate-depleted beetroot juice

### Explosive strength

There was no interaction effect (condition x time) or main effect of condition or time on upper body explosive strength as assessed by seated medicine ball throw, or on lower body explosive strength as assessed by standing long jumps (*P* > 0.05, Table 5).

**Table 5:** Explosive strength performance before and after the YYIR1 test following acute beetroot juice ingestion

Variable	PL		BR	
	Pre	Post	Pre	Post
Seated medicine ball throw distance (m)	4.45 ± 0.48	4.35 ± 0.48	4.41 ± 0.38	4.49 ± 0.47
Standing long jump distance (m)	1.72 ± 0.27	1.72 ± 0.26	1.77 ± 0.25	1.73 ± 0.28

BR, nitrate-rich beetroot juice; PL, nitrate-depleted beetroot juice

### YYIR1 performance

The distance covered during the YYIR1 and RPE score are displayed in Table 6. The total distance covered in the YYIR1 was not significantly different in BR compared to PL (*P* = 0.494, *d*_*z*_ = 0.18). There were no differences in RPE at the end of the YYIR1 between conditions (*P* > 0.05).

**Table 6:** Performance outcomes for the YYIR1 test following acute beetroot juice ingestion

Variable	PL	BR
Total distance covered (m)	355 ± 163	368 ± 184
Rate of perceived exertion	17 ± 1	17 ± 1

BR, nitrate-rich beetroot juice; PL, nitrate-depleted beetroot juice

## Discussion

The main novel findings of the present study were that acute BR did not shift the oral microbial composition or improve sprint performance, maximal or explosive strength, or cognitive flexibility before, during, and after YYIR1 testing, or exhaustive YYIR1 performance. These findings do not support our hypotheses and indicate that an acute dose of NO_3_^−^ was not effective at improving a battery of performance tests in women team-sport players.

### The influence of BR on nitric oxide bioavailability and the oral microbiome

Plasma [NO_3_^−^] and [NO_2_^−^] were increased following the acute ingestion of BR compared to PL which aligns with previous studies in males (Nyakayiru et al. 2017; Wylie et al. 2013b) and women (Glaister et al. 2015; Lane et al. 2014; Wickham et al. 2019). While the elevation in [NO_3_^−^] and [NO_2_^−^] following NO_3_^−^ ingestion is important, emerging evidence suggests that S-nitrosothiols are an independent NO reservoir and may be involved in dietary NO_3_^−^ metabolism and actions, which may be important for future studies to consider (Abu-Alghayth et al. 2021; Wei et al. 2024).

Acute dietary NO_3_^−^ ingestion did not influence oral microbial communities. Specifically, the relative abundances of the most abundant phyla (*Firmicutes* and *Proteobacteria*), and some genera (*Haemophilus*) were not altered by acute NO_3_^−^ ingestion compared to PL. Previous studies note that NO_3_^−^ ingestion (12 mmol NO_3_^−^ for 7–10 days) increased the relative abundance of *Neisseria* and *Rothia* (Burleigh et al. 2019; Vanhatalo et al. 2018, 2021)–bacterial species with known NO_3_^−^ reducing capacity (Hyde et al. 2014), which have been associated with greater NO bioavailability after NO_3_^−^ ingestion (Vanhatalo et al. 2018). However, we did not identify either of these taxa in these buccal samples, which may be due to methodological limitations. Furthermore, there were no differences in global oral microbial composition between BR and PL which contrasts previous findings that reported distinct microbial communities between BR and PL following NO_3_^−^ supplementation (Vanhatalo et al. 2018, 2021).

Notably, our study analyzed the buccal cell microbiome, while previous studies in this field analyzed tongue swabs or saliva samples (Burleigh et al. 2019; Hyde et al. 2014; Kapil et al. 2013; Vanhatalo et al. 2018, 2021). This is an important discrepancy in methodology when comparing our study to previous studies as the buccal microbiome is a distinct oral microbial niche, likely to be represented by bacteria that have the ability to adhere to the inner cheek (Santacroce et al. 2023; Wang et al. 2022). Though some of these buccal-associated bacteria will be found in saliva, a difference in the composition across these sample types should be expected. Additionally, we analyzed the V3–V4 region of the 16S gene, which is common for the analysis of the buccal microbiome (Wang et al. 2022). In contrast, previous studies compared V1–V3 or V3–V5 for the analysis of saliva samples (Vanhatalo et al. 2018, 2021), and variable regions of the 16S gene can yield different taxonomic classifications (López-Aladid et al. 2023).

Other possible reasons for the discrepancy in our findings relative to other studies are that longer supplementation durations may be required to alter the oral microbiome (Burleigh et al. 2019; Vanhatalo et al. 2018, 2021), and/or that host-microbiome interactions may be population specific (Minty et al. 2021; Yang et al. 2019). While we exclusively included women, previous studies included healthy young men (Burleigh et al. 2019), a mixture of healthy young men and women (Kapil et al. 2013, 2018), and older men and women (Vanhatalo et al. 2021). Future research is advised to investigate the impact of dosing strategy on bacterial taxa and whether NO_3_^−^-induced microbial adaptations translate into meaningful physiological and performance effects in various populations.

### The influence of BR on exercise performance

An original contribution of the current study is the assessment of numerous aspects of exercise performance (i.e., sprints, upper and lower body maximal, and explosive strength) in an unfatigued and fatigued state before and after the YYIR1 in women. There was no effect of BR on split times or response times during 20-m sprint, and maximal and explosive strength before and after the YYIR1. These data contrast with our hypotheses, but are consistent with the previous studies that examined NO_3_^−^ ingestion on sprint performance (López-Samanes et al. 2022, 2023), maximal isokinetic handgrip strength (López-Samanes et al. 2022, 2023), and lower body explosive strength (López-Samanes et al. 2023) in women team-sport players. Another original contribution of the current study is that we examined YYIR1 performance in women and found that there was no significant influence of BR on total distance covered. This is in contrast to several studies conducted exclusively in men which observed a 3.9–4.2% improvement in YYIR1 performance after NO_3_^−^ supplementation (Nyakayiru et al. 2017; Thompson et al. 2016; Wylie et al. 2013b)—although a 14% improvement was recently observed in recreationally active men (Esen et al. 2022). Based on data from studies with men, it is possible that longer term supplementation may be more efficacious for YYIR1 performance.

Collectively, acute NO_3_^−^ ingestion was ineffective at eliciting ergogenic effects in sprinting, strength, and aerobic performance in women. It is possible that sex differences in fiber-type composition contributed to the lack of effect. Indeed, women could have a more oxidative phenotype and thus relatively less type II muscle fibers compared to men (Haizlip et al. 2015), which could compromise the ergogenic potential of NO_3_^−^ supplementation (Ferguson et al. 2013; Hernández et al. 2012). Another possible explanation is that a lower dose of NO_3_^−^ would be more efficacious in women given that estrogen is linked to increased endothelial NO synthase expression and thus NO synthesis (Yang et al. 2000), and that recent data suggest that higher NO_3_^−^ doses, and thus, potentially higher NO bioavailability, may result in worse exercise performance, although this study was conducted in older individuals (Gallardo et al. 2021). Indeed, we observed that plasma [NO_2_^−^] increased by ~ 50% to ~ 470% following NO_3_^−^ supplementation, and therefore, it is possible that interindividual variation in the elevation of NO bioavailability post-supplementation could have contributed to the lack of effects observed. However, further research is required to understand the impact of the magnitude of elevation in NO bioavailability on the efficacy of dietary NO_3_^−^ supplementation.

### The effect of BR on neuropsychological outcomes

Response time (i.e., a proxy measure of central nervous system processing speed) following a randomized visuo-auditory stimulus at the start of the 20-m sprint was not influenced by the YYIR1 or BR. Our results contrast with previous studies conducted in men, which reported improved reactive agility response times (Rogers et al. 2022), Stroop test reaction time (Thompson et al. 2015), and simple reaction (Gilchrist et al. 2014) following NO_3_^−^ supplementation. However, cognitive flexibility scores improved over time in both conditions, which could indicate that a learning effect occurred despite that practice tests were performed prior to each test. Cognitive performance was assessed via cognitive flexibility testing using a norm-referenced, design fluency test previously shown to be predictive of sporting success in team-sports athletes (Vestberg et al. 2012, 2017). Thus, we captured robust cognitive data that may be generalizable to the sport performance domain (e.g., creativity, decision-making, and adaptability) as opposed to specific (or arbitrary) neuropsychological outcomes. It is possible that high NO_3_^−^ doses or longer supplementation periods may be required to elicit beneficial cognitive effects, although it is notable that an acute NO_3_^−^ dose (5.5 mmol NO_3_^−^) was effective at improving serial 3’s subtraction task (i.e., measure of attention, working memory, and sequencing) in healthy adults (Wightman et al. 2015).

### Limitations

The sequence counts in the microbiome samples were low, which may be due to technical limitations. It could be possible that sequencing to a greater depth would increase the likelihood of identifying more taxa at the genus level. Furthermore, due to logistical constraints, we did not plan the timing of experimental visits to coincide with a particular phase of the menstrual cycle (e.g., early follicular phase, late follicular phase, or mid luteal phase), or compare physiological responses to NO_3_^−^ supplementation between menstrual cycle phases. However, the same phase was tested between each condition within participants. Moreover, due to financial constraints, we did not measure sex hormone concentrations (estradiol and progesterone), so it was not possible to verify hormonal status on experimental testing days. We acknowledge that there is a distinct hormonal milieu in each of these menstrual cycle phases which may influence the efficacy of NO_3_^−^ supplementation, and thus, further work is needed to ascertain whether there are differences in responsiveness to NO_3_^−^ supplementation across the menstrual cycle. Furthermore, future studies may consider implementing questionnaires to verify blinding procedures to ensure that participants did not detect differences between supplements (Poulios et al. 2018).

## Conclusions

Acute NO_3_^−^-rich beetroot juice ingestion increased plasma [NO_3_^−^] and [NO_2_^−^] compared to PL in women team-sport players. However, BR ingestion did not influence the oral microbial composition at the global level or in relative abundance. Moreover, BR did not impact physical or cognitive performance across a battery of tests before, during, and after the YYIR1 intermittent running test. Therefore, these data indicate that an acute dose of dietary NO_3_^−^ was not effective at improving high-intensity exercise performance or cognitive flexibility in women under these circumstances.

## Supplementary Information

Below is the link to the electronic supplementary material.

Supplementary file1 (PDF 25 KB)

Supplementary file2 (PDF 26 KB)

Supplementary file3 (XLSX 21 KB)

## Data Availability

The raw data supporting the conclusions of this article will be made available by the authors, without undue reservation.
