# Supplementary material for: Acute inorganic nitrate ingestion does not impact oral microbial composition, cognitive function, or high-intensity exercise performance in female team-sport athletes
Source: Eur J Appl Physiol. 2024 Jul 17;124(12):3511–25. doi: 10.1007/s00421-024-05552-w (PMC11568988; doi:10.1007/s00421-024-05552-w)
Supplement: Supplementary file 1 — Supplementary file1 (PDF 25 KB) [file 421_2024_5552_MOESM1_ESM.pdf]

```
#After DADA2 Processing
RT01 <- phyloseq(otu_table(seqtab.nochim, taxa_are_rows=FALSE),tax_table(taxa), sample_data(samdata))

#add phylo tree
RT01 <- subset_taxa(RT01, Kingdom == 'Bacteria') #keep only bacterial reads
random_tree = rtree(ntaxa(RT01), rooted=TRUE, tip.label=taxa_names(ps)) #produce a rooted tree
#Warning message:
  #In rtree(ntaxa(Tan_data), rooted = TRUE, tip.label = taxa_names(ps)) :
  #vector 'tip.label' longer than 'n': was shorten
RT01_treed = merge_phyloseq(RT01, random_tree)

#decontaminate
contamdf.prev <- isContaminant(RT01_treed, method="prevalence", neg="is.neg", threshold=0.5)
#Warning message:
  #In isContaminant(RT01_treed, method = "prevalence", neg = "is.neg",  :
                  #Removed 1 samples with zero total counts (or frequency).
table(contamdf.prev$contaminant)
head(which(contamdf.prev$contaminant))
#[1]  464 2188
RT01_decontam <- prune_taxa(!contamdf.prev$contaminant, RT01_treed)
RT01_decontam
#phyloseq-class experiment-level object
#otu_table()   OTU Table:         [ 2558 taxa and 30 samples ]
#sample_data() Sample Data:       [ 30 samples by 4 sample variables ]
#tax_table()   Taxonomy Table:    [ 2558 taxa by 6 taxonomic ranks ]
#phy_tree()    Phylogenetic Tree: [ 2558 tips and 2557 internal nodes ]


#RT01 rarefaction and removal of low read samples

sort(phyloseq::sample_sums(RT01_decontam)) #sort by read counts
#Blank     Pos   S11V3B S11MPV4A   S1V4A2   S18V3B   S15V4A   S15V3B    S2V3A   S1V3B2   S5V3A3
#0       0      47     2223     3071     3545     3555     4339     4680     4973     5296
#S10V4B   S20V3A    S5V4B   S6V4A3   S16V4B  S3CRV3B S14MHV4B   S9V4B2   S18V4A    S2V4B
S16V4A
#5446     5451     5516     5831     6036     6135     6179     8164     9185     9253     9261
#S14MHV3A  S3CRV3A  S12V4A2   S6V3B3   S10V3A  S12V3B2   S20V4B   S9V3A2
#9613    10191    10340    11942    11985    14682    15083    16839

Tan <- subset_samples(RT01_decontam, sample_sums(RT01_decontam) > 1000)
Tan
#phyloseq-class experiment-level object
#otu_table()   OTU Table:         [ 2558 taxa and 27 samples ]
#sample_data() Sample Data:       [ 27 samples by 4 sample variables ]
#tax_table()   Taxonomy Table:    [ 2558 taxa by 6 taxonomic ranks ]
#phy_tree()    Phylogenetic Tree: [ 2558 tips and 2557 internal nodes ]
```

RT01_decontam
#phyloseq-class experiment-level object
#otu_table() OTU Table: [2558 taxa and 30 samples]
#sample_data() Sample Data: [30 samples by 4 sample variables]
#tax_table() Taxonomy Table: [2558 taxa by 6 taxonomic ranks]
#phy_tree() Phylogenetic Tree: [2558 tips and 2557 internal nodes] #compare new data to old

Tan_R = rarefy_even_depth(Tan, rngseed=1, sample.size = 2223, replace = FALSE, verbose = TRUE)

RT01_rarefied
#phyloseq-class experiment-level object
#otu_table() OTU Table: [2160 taxa and 27 samples]
#sample_data() Sample Data: [27 samples by 4 sample variables]
#tax_table() Taxonomy Table: [2160 taxa by 6 taxonomic ranks]
#phy_tree() Phylogenetic Tree: [2160 tips and 2159 internal nodes]

#RT01 relative abundance
top100 <- names(sort(taxa_sums(Tan), decreasing=TRUE))[1:100]
> top100_trs <- transform_sample_counts(Tan, function(OTU) OTU/sum(OTU))
> top100_prune <- prune_taxa(top100, top100_trs)
> merge = merge_samples(top100_prune, "Beetroot")
> sample_data(merge)$Beetroot<- levels(sample_data(Tan)$Beetroot)
> merge.100 = transform_sample_counts(merge, function(x) 100 * x/sum(x))
> p1 <- plot_bar(merge.100, "Beetroot", "Abundance", "Phylum") #change to p2 and genus, below is final code to make graphs, change that to p1 for phylum graph
p2 + geom_bar(aes(color=Genus, fill=Genus), stat="identity", position='stack') + xlab("Supplementation") + ylab("Percent Abundnace") + ggtitle("Effect of Beetroot on Relative Genus Abundance of the Oral Microbiome") + theme_bw() + theme(axis.text.x=element_text(size=12), axis.text.y=element_text(size=14),axis.title=element_text(size=16), title = element_text(size=16))
#RT01 alpha
pal = c("#E69F00", "#0072B2") #color palette
richness <- plot_richness(tan_R, "Beetroot", measures=c("Shannon", "Chao1"), color="Beetroot") + geom_boxplot() + theme_bw() + scale_colour_brewer("Beetroot", palette="Set1") + scale_fill_brewer("Beetroot", palette="Set1") + xlab("Supplementation with Beetroot") + ylab("Diversity Index") + ggtitle("Effect of Beetroot on Alpha Diversity of the Oral Microbiome") + theme(axis.text.x=element_text(size=12), axis.text.y=element_text(size=14),axis.title=element_text(size=16), title = element_text(size=16)) + geom_point(size = 5) + scale_color_manual(values=pal)
resultsS <- estimate_richness(tan_R, measures = 'Chao1') #change all to shannon for shannon
d = sample_data(tan_R) #variables are in your sample data
resS <- cbind(resultsS, d)
resS
shapiro.test(resS$Chao1)
aov<- aov(Chao1~Beetroot, data=resS)
summary(aov)
#both yielded non significant findings. Shapiro wilk p>0.05, anovas p>0.05.

#RT01 - beta diversity

```
ord <- ordinate(tan_R, "PCoA", "wunifrac") #change to unifrac for unweighted
plot_ord <- plot_ordination(tan_R, ord, type="samples", color="Beetroot", title="Beta Diversity Across Sample
Types") + geom_point=(size=5) #use unweighted unifrac ord for that plot
#ran betadisper (p>0.05), ran permanova for both too, p>0.05. Not signfificant.
#betadisper code
distance = distance(tan_R, "UniFrac")
> groups <- sample_data(tan_R)$Beetroot
> mod <- betadisper(distance, groups)
> permutest(mod)
#permanova code
dist = distance(tan_R, "wunifrac")
> adonis(dist ~ Beetroot, as(sample_data(tan_R), "data.frame"))
#unweighted
plot_orduw + xlab("PC 1 [19.9%]") + ylab("PC 2 [11.1%]") + ggtitle("Effect of Beetroot on Beta Diversity of the
Oral Microbiome (Unweighted Unifrac)") + theme_bw() + theme(axis.text.x=element_text(size=12),
axis.text.y=element_text(size=14),axis.title=element_text(size=14), title = element_text(size=16),
                                                                                                    +
legend.title = element_text(size = 14),
                                                                                                    +
legend.text = element_text(size = 12)
#weighted unifrac
plot_ord + xlab("PC 1 [35.6%]") + ylab("PC 2 [18.2%]") + ggtitle("Effect of Beetroot on Beta Diversity of the
Oral Microbiome (Weighted Unifrac)") + theme_bw() + theme(axis.text.x=element_text(size=12),
axis.text.y=element_text(size=14),axis.title=element_text(size=14), title = element_text(size=16),
                                                                                                  +
legend.title = element_text(size = 14),

 #Maaslin Stats to check for differential ASVs
  analysisdata_pruned_abund <- microbiome::transform(Tan,
                                          transform = "compositional",
                                          target = "OTU", shift = 0,
                                          scale = 1)
  ASVs <- as.data.frame(analysisdata_pruned_abund@otu_table) #convert to dataframe
  input_data <- as.data.frame(t(ASVs))
  meta <- as.matrix(analysisdata_pruned_abund@sam_data)
  meta <- as.data.frame(meta) #make datafram
  library(Maaslin2)

  fit_data = Maaslin2(
    input_data = ASVs,
    input_metadata = meta,
    output = "/Data_Sets/Tan_Seqs/RT01_May2023", #whatever folder you've been working in.
    fixed_effects = c("Beetroot"), plot_heatmap = TRUE)
#There are no associations to plot! - thus nothing significant.
```
