# Supplementary material for: Acute inorganic nitrate ingestion does not impact oral microbial composition, cognitive function, or high-intensity exercise performance in female team-sport athletes
Source: Eur J Appl Physiol. 2024 Jul 17;124(12):3511–25. doi: 10.1007/s00421-024-05552-w (PMC11568988; doi:10.1007/s00421-024-05552-w)
Supplement: Supplementary file 2 — Supplementary file2 (PDF 26 KB) [file 421_2024_5552_MOESM2_ESM.pdf]

Supplementary Materials Online Resource 2: oral microbiome analysis.

Title: Acute inorganic nitrate ingestion does not impact oral microbial composition, cognitive function, or high-intensity exercise performance in female team-sport athletes
Journal: European Journal of Applied Physiology
Author names: Rachel Tan, Courtney Merrill, Chandler Riley, Maya Hammer, Ryan Kenney, Alyssa Riley, Jeffrey Li, Alexandra Zink, Sean Karl, Katherine Price, Luka Sharabidze, Samantha Rowland, Stephen Bailey, Leah Stiemsma, Adam Pennell
Corresponding author email: rachel.tan@pepperdine.edu

sample_name	library_ID	title	library_strategy	library_source	library_selection	library_layout	platform	instrument_model	design_description	filetype	filename	filename2	filename3	filename4	assembly	fasta_file
1_oral	1_oral	16S amplicon of buccal cells	AMPLICON	METAGENOMIC	PCR	paired	ILLUMINA	Illumina iSeq 100	16S PCR	fastq	S1V3B2_S1_L001_R1_001.fastq.gz	S1V3B2_S1_L001_R2_001.fastq.gz				
2_oral	2_oral	16S amplicon of buccal cells	AMPLICON	METAGENOMIC	PCR	paired	ILLUMINA	Illumina iSeq 100	16S PCR	fastq	S1V4A2_S2_L001_R1_001.fastq.gz	S1V4A2_S2_L001_R2_001.fastq.gz				
3_oral	3_oral	16S amplicon of buccal cells	AMPLICON	METAGENOMIC	PCR	paired	ILLUMINA	Illumina iSeq 100	16S PCR	fastq	S2V3A_S3_L001_R1_001.fastq.gz	S2V3A_S3_L001_R2_001.fastq.gz				
4_oral	4_oral	16S amplicon of buccal cells	AMPLICON	METAGENOMIC	PCR	paired	ILLUMINA	Illumina iSeq 100	16S PCR	fastq	S2V4B_S4_L001_R1_001.fastq.gz	S2V4B_S4_L001_R2_001.fastq.gz				
5_oral	5_oral	16S amplicon of buccal cells	AMPLICON	METAGENOMIC	PCR	paired	ILLUMINA	Illumina iSeq 100	16S PCR	fastq	S3CRV3A_S5_L001_R1_001.fastq.gz	S3CRV3A_S5_L001_R2_001.fastq.gz				
6_oral	6_oral	16S amplicon of buccal cells	AMPLICON	METAGENOMIC	PCR	paired	ILLUMINA	Illumina iSeq 100	16S PCR	fastq	S3CRV3B_S6_L001_R1_001.fastq.gz	S3CRV3B_S6_L001_R2_001.fastq.gz				
7_oral	7_oral	16S amplicon of buccal cells	AMPLICON	METAGENOMIC	PCR	paired	ILLUMINA	Illumina iSeq 100	16S PCR	fastq	S5V3A3_S7_L001_R1_001.fastq.gz	S5V3A3_S7_L001_R2_001.fastq.gz				
8_oral	8_oral	16S amplicon of buccal cells	AMPLICON	METAGENOMIC	PCR	paired	ILLUMINA	Illumina iSeq 100	16S PCR	fastq	S5V4B_S8_L001_R1_001.fastq.gz	S5V4B_S8_L001_R2_001.fastq.gz				

9_oral 9_oral 16S amplicon of buccal cells AMPLICON METAGENOMIC
PCR paired ILLUMINA Illumina iSeq 100 16S PCR
fastq S6V4A3_S9_L001_R1_001.fastq.gz
S6V4A3_S9_L001_R2_001.fastq.gz
10_oral 10_oral 16S amplicon of buccal cells AMPLICON METAGENOMIC
PCR paired ILLUMINA Illumina iSeq 100 16S PCR
fastq S6V3B3_S10_L001_R1_001.fastq.gz
S6V3B3_S10_L001_R2_001.fastq.gz
11_oral 11_oral 16S amplicon of buccal cells AMPLICON METAGENOMIC
PCR paired ILLUMINA Illumina iSeq 100 16S PCR
fastq S9V3A2_S11_L001_R1_001.fastq.gz
S9V3A2_S11_L001_R2_001.fastq.gz
12_oral 12_oral 16S amplicon of buccal cells AMPLICON METAGENOMIC
PCR paired ILLUMINA Illumina iSeq 100 16S PCR
fastq S9V4B2_S12_L001_R1_001.fastq.gz
S9V4B2_S12_L001_R2_001.fastq.gz
13_oral 13_oral 16S amplicon of buccal cells AMPLICON METAGENOMIC
PCR paired ILLUMINA Illumina iSeq 100 16S PCR
fastq S10V3A_S13_L001_R1_001.fastq.gz
S10V3A_S13_L001_R2_001.fastq.gz
14_oral 14_oral 16S amplicon of buccal cells AMPLICON METAGENOMIC
PCR paired ILLUMINA Illumina iSeq 100 16S PCR
fastq S10V4B_S14_L001_R1_001.fastq.gz
S10V4B_S14_L001_R2_001.fastq.gz
15_oral 15_oral 16S amplicon of buccal cells AMPLICON METAGENOMIC
PCR paired ILLUMINA Illumina iSeq 100 16S PCR
fastq S11MPV4A_S15_L001_R1_001.fastq.gz
S11MPV4A_S15_L001_R2_001.fastq.gz
16_oral 16_oral 16S amplicon of buccal cells AMPLICON METAGENOMIC
PCR paired ILLUMINA Illumina iSeq 100 16S PCR
fastq S20V4B_S28_L001_R1_001.fastq.gz
S20V4B_S28_L001_R2_001.fastq.gz
17_oral 17_oral 16S amplicon of buccal cells AMPLICON METAGENOMIC
PCR paired ILLUMINA Illumina iSeq 100 16S PCR
fastq S12V4A2_S17_L001_R1_001.fastq.gz
S12V4A2_S17_L001_R2_001.fastq.gz
18_oral 18_oral 16S amplicon of buccal cells AMPLICON METAGENOMIC
PCR paired ILLUMINA Illumina iSeq 100 16S PCR
fastq S12V3B2_S18_L001_R1_001.fastq.gz
S12V3B2_S18_L001_R2_001.fastq.gz
19_oral 19_oral 16S amplicon of buccal cells AMPLICON METAGENOMIC
PCR paired ILLUMINA Illumina iSeq 100 16S PCR
fastq S14MHV3A_S19_L001_R1_001.fastq.gz
S14MHV3A_S19_L001_R2_001.fastq.gz
20_oral 20_oral 16S amplicon of buccal cells AMPLICON METAGENOMIC
PCR paired ILLUMINA Illumina iSeq 100 16S PCR
fastq S14MHV4B_S20_L001_R1_001.fastq.gz
S14MHV4B_S20_L001_R2_001.fastq.gz
21_oral 21_oral 16S amplicon of buccal cells AMPLICON METAGENOMIC
PCR paired ILLUMINA Illumina iSeq 100 16S PCR

fastq	S15V4A_S21_L001_R1_001.fastq.gz
S15V4A_S21_L001_R2_001.fastq.gz
22_oral	22_oral	16S amplicon of buccal cells	AMPLICON	METAGENOMIC
PCR	paired	ILLUMINA	Illumina iSeq 100	16S PCR
fastq	S15V3B_S22_L001_R1_001.fastq.gz
S15V3B_S22_L001_R2_001.fastq.gz
23_oral	23_oral	16S amplicon of buccal cells	AMPLICON	METAGENOMIC
PCR	paired	ILLUMINA	Illumina iSeq 100	16S PCR
fastq	S16V4A_S23_L001_R1_001.fastq.gz
S16V4A_S23_L001_R2_001.fastq.gz
24_oral	24_oral	16S amplicon of buccal cells	AMPLICON	METAGENOMIC
PCR	paired	ILLUMINA	Illumina iSeq 100	16S PCR
fastq	S16V4B_S24_L001_R1_001.fastq.gz
S16V4B_S24_L001_R2_001.fastq.gz
25_oral	25_oral	16S amplicon of buccal cells	AMPLICON	METAGENOMIC
PCR	paired	ILLUMINA	Illumina iSeq 100	16S PCR
fastq	S18V3B_S25_L001_R1_001.fastq.gz
S18V3B_S25_L001_R2_001.fastq.gz
26_oral	26_oral	16S amplicon of buccal cells	AMPLICON	METAGENOMIC
PCR	paired	ILLUMINA	Illumina iSeq 100	16S PCR
fastq	S18V4A_S26_L001_R1_001.fastq.gz
S18V4A_S26_L001_R2_001.fastq.gz
27_oral	27_oral	16S amplicon of buccal cells	AMPLICON	METAGENOMIC
PCR	paired	ILLUMINA	Illumina iSeq 100	16S PCR
fastq	S20V3A_S27_L001_R1_001.fastq.gz
S20V3A_S27_L001_R2_001.fastq.gz
